# Root Interactions in a Maize/Soybean Intercropping System Control Soybean Soil-Borne Disease, Red Crown Rot

**DOI:** 10.1371/journal.pone.0095031

**Published:** 2014-05-08

**Authors:** Xiang Gao, Man Wu, Ruineng Xu, Xiurong Wang, Ruqian Pan, Hye-Ji Kim, Hong Liao

**Affiliations:** 1 State Key Laboratory for Conservation and Utilization of Subtropical Agro-bioresources, Root Biology Center, South China Agricultural University, Guangzhou, China; 2 Department of Tropical Plants and Soil Sciences, College of Tropical Agriculture and Human Resources, University of Hawaii at Manoa, Honolulu, Hawaii, United States of America; University of Wisconsin-Milwaukee, United States of America

## Abstract

**Background:**

Within-field multiple crop species intercropping is well documented and used for disease control, but the underlying mechanisms are still unclear. As roots are the primary organ for perceiving signals in the soil from neighboring plants, root behavior may play an important role in soil-borne disease control.

**Principal Findings:**

In two years of field experiments, maize/soybean intercropping suppressed the occurrence of soybean red crown rot, a severe soil-borne disease caused by *Cylindrocladium parasiticum* (*C. parasiticum*). The suppressive effects decreased with increasing distance between intercropped plants under both low P and high P supply, suggesting that root interactions play a significant role independent of nutrient status. Further detailed quantitative studies revealed that the diversity and intensity of root interactions altered the expression of important soybean *PR* genes, as well as, the activity of corresponding enzymes in both P treatments. Furthermore, 5 phenolic acids were detected in root exudates of maize/soybean intercropped plants. Among these phenolic acids, cinnamic acid was released in significantly greater concentrations when intercropped maize with soybean compared to either crop grown in monoculture, and this spike in cinnamic acid was found dramatically constrain *C. parasiticum* growth *in vitro*.

**Conclusions:**

To the best of our knowledge, this study is the first report to demonstrate that intercropping with maize can promote resistance in soybean to red crown rot in a root-dependent manner. This supports the point that intercropping may be an efficient ecological strategy to control soil-borne plant disease and should be incorporated in sustainable agricultural management practices.

## Introduction

Reducing disease severity through increasing the within-field multiple crop species, intercropping has been widely reported in the literature and commercially implemented [1]. For example, disease-susceptible rice varieties have been grown in combination with resistant varieties to achieve higher yield and greatly suppress blast severity compared to growth in monoculture [2]. Also, growing mixtures of resistant oats and susceptible barley results in decreased stem rust severity, and thereby increases yield [3]. However, the mechanisms underlying these heterogeneous genotype and crop species effects on disease control remain unclear.

Soil-borne diseases are caused by phytopathogenic microbes in the soil, which may damage plants upon penetration of the root or basal stem [3]. With a potential to seriously harm the agroecosystem, and with broad geographic ranges, soil-borne diseases can significantly impact global food production [4]. Furthermore, eradication of the pathogen is difficult as long as soil-borne disease pathogens accumulate in cultivated soils. To date, effective methods to control soil-borne diseases remain lacking. Therefore, the simple and economic alternative of agricultural management is often a preferred method for preventing crop losses caused by soil-borne diseases. It has long been recognized that crop heterogeneity suppresses soil-borne disease infection and appears to be a more promising solution than petrochemical-based pesticide application. Among the successful examples of this strategy are inhibition of tomato bacterial wilt caused by *Pseudomonas solanacearum* in a Chinese chive-tomato intercropping system [5], suppression of Fusarium wilt in rice with watermelon intercropping [6], [7], and alleviation of tomato early blight disease by intercropping with marigold [8].

One potential target for control through crop heterogeneity is red crown rot, which is one of the most severe soil-borne diseases for plants. It is caused by the fungus *Cylindrocladium parasiticum* (*C. parasiticum*, teleomorph *Calonectria ilicicola*) and is named after the reddish brown basal stalk tissue of infected plants [9]–[11]. This pathogen has a wide host range, and red crown rot has been found in many countries with severe crop losses possible throughout its distribution. Soybean (*Glycine max* L. Merr.), an economically important host of red crown rot, is a major cash crop and has been planted world wide. In South China, estimates for soybean yield loss due to red crown rot range to as high as 50% [11]. At present, there are no effective pesticides to control red crown rot.

Intercropping has been widely used in Asia, Latin America and Africa to enhance crop productivity through improved nutrient uptake, enhanced land equivalent ratio (LER), increased yield, and better disease control [12], [13]. Legume/cereal intercropping is the most common intercropping system, which not only allows crops to take up more nutrients than in monoculture, but also reduces disease occurrence [14], [15]. However, little attention has been paid to the belowground environment of the plant root systems. As the main organ to contact the soil and perceive signals from neighboring plants [16], root function and behavior should be particularly important in soil-borne disease control. In one case, Fang et al. [17] reported that root interactions in the maize/soybean intercropping can integrate responses to P status and root behaviors among neighboring plants, and thereby impact maize P nutrition. A previous study by the current authors found that co-inoculation with rhizobia and mycorrhizal fungi can inhibit soybean red crown rot [18]. However, uncertainty remains about what happens when only mycorrhizal fungi, which favor maize over soybean, are introduced. In this study, effects of maize intercropping with soybean on red crown rot in soybean were studied in two years of field experiments. Further in-door experiments were then carried out to evaluate the underlying mechanisms for inhibition of soybean red crown rot by intercropping with maize.

## Methods and Materials

1. The Boluo (E114.28°, N23.18°) field site is the experimental base of the South China Agricultural University Root Biology Center. Therefore, the authority that issued the permit for this location is South China Agricultural University.
2. No specific permissions were required for this location. I am here to confirm that the field studies did not involve endangered or protected species.

### Field trials

Field experiments were carried out on acidic lateritic red soils at the Boluo field site (E114.28°, N23.18°) in the Guangdong Province of China from July to October in 2009 and 2010 with the average day/night temperature around 25–33°C and monthly rainfall around 150 mm. Basic soil chemical characteristics were as follows: pH, 5.37; organic matter, 17.63 g kg^−1^; available P (Bray I method), 15.68 mg P kg^−1^; available N, 86.64 mg N kg^−1^; available K, 75.28 mg K kg^−1^. The field site had a history of up to 10 years of continuous soybean cultivation, in which severe soybean red crown rot (RCR) damage occurred [11]. Two years of field trials were conducted in a split-block design with two P supplies (high P and low P), and four cultivation modes (soybean monoculture, maize monoculture, two maize/soybean intercropping systems) for a total of 8 treatments (Figure A in File S1). High P (HP) was implemented by adding calcium superphosphate (SSP) at the rate of 80 kg P_2_O_5_ ha^−1^, and low P (LP) plots had no P fertilizer added. The two maize/soybean intercropping systems differed in planting distances between soybean and maize plants, which were 20 cm for ISC1 and 5 cm for ISC2. Urea and KCl were added to all plots as 80 kg N ha^−1^ and 60 kg K ha^−1^ for supplementing N and K supplies. Each treatment had four replicates with a total of 32 plots (i.e. 2 P × 4 mode × 4 replicates). The planting area of each plot was 54 m^2^. Soybean (*Glycine max*) variety HN89 and maize (*Zea mays*) variety ZD958 were employed in the field experiment.

Seventy days after planting, 100 plants were selected from each plot to investigate the severity of RCR. Disease incidence and severity caused by *C. parasiticum* were determined according to Gao et al. [18]. Disease incidence was defined as the percentage of infected subterranean stems. Disease severity was recorded on a 0–5 scale: i.e., 0, no visible symptoms; 1, small necrotic lesions on the subterranean stem; 2, necrotic lesions extending around the subterranean stem, 3, necrotic lesions extending to the soil surface; 4, severe necrosis on the subterranean stem and roots, as well as severe chlorosis appearing in leaves; 5, plant death. The disease index was summarized within each plot as{[(n_1_×1)+(n_2_×2)+(n_3_×3)+…+(n_N_×N)]/[N×(n_1_+n_2_+n_3_…+n_N_)]}×100, where n_1_…n_N_ was the number of subterranean stems in each of the respective disease category, and N was the highest disease severity score [2], [18].

Seventy days after planting, two representative healthy soybean plants from each plot were harvested for measuring root length and symbiotic traits. Total root length in the 20-cm upper soil layer of the 5-cm strip between intercropped plants was measured to represent the intensity of interacting roots between the two crop species in the field as described by Fang et al. [16]. In short, trenches were dug by shoveling between rows of intercropped plants. The walls were carefully scraped with a screwdriver to reveal the tips of the roots. Plastic transparent sheets (25×30 cm) were positioned adjacent to the exposed soil wall. The roots in the 20 cm upper soil layer were marked on the sheets, and the root length in the 5 cm section between the two plants was quantitatively measured with root image analysis software (WinRhizo Pro, Régent Instruments). Arbuscular mycorrhizal fungi (AMF) colonization rates and nodule numbers on soybean roots were measured according to Wang et al. [19].

At maturity, soybean biomass and yield were average for 20 healthy and 20 infected plants within each soybean monoculture plot. Growth reduction was calculated as: reduction (%)  =  (yield/biomass of healthy plants - yield/biomass of infected plants) yield/biomass of healthy plants × 100. This value was determined for each of the four replicates separately.

### Root barrier sand culture

Root barrier sand culture experiments were conducted in the greenhouse of Root Biology Center of South China Agricultural University in 2011 using soybean variety HN89 and maize variety ZD958. *Cylindrocladium parasiticum* (*C. parasiticum*, GenBank Accession No. GU073284) was isolated from infected soybean roots at the Boluo field site and used as the pathogen inoculant [11]. The sand culture experiment consisted of 3 factors (P level, root barrier and pathogen inoculation), including two P levels, three root barrier arrangements and two pathogen inoculations, for a total of 12 treatments. There were 6 replicates for each treatment. The two P levels were HP (500 µM P) and LP (15 µM P). Plastic pots with two equal compartments were used to provide three types of root barriers as illustrated in Figure A in File S1 and described as follows: solid barrier to eliminate root interaction and exudates movement, a nylon mesh barrier (30 µm pores) to prevent interspecies root intermingling while permitting root exudates exchange, and no barrier to allow roots and exudates to completely interact. Soybean plants were inoculated with *C. parasiticum* infected wheat seeds or sterilized media as a control before sowing. Wheat seeds of infected *C. parasiticum* were obtained from 14-day-old media inoculated with *C. parasiticum*. The spore concentration was determined using a hemocytometer and adjusted to 1×10^5^ spores per mL [18]. Before sowing, infected wheat seeds were placed at depth of 5 cm in sand culture pots. Two independent biological experiments were conducted, with very similar results between experiments, so only the results from one experiment were presented.

Plants were irrigated daily using modified 1/2 strength Hoagland nutrient solution with either of the two P additions listed above [20]. Sixty days after planting, incidence and severity of red crown rot was measured as described above. In order to determine the root infection by *C. parasiticum*, root colony forming units (CFUs) were measured from sand culture plants. Randomly selected soybean roots were cut into 1 cm segments and washed thoroughly and surface-sterilized with 0.5% (v/v) NaClO for 3 min, rinsed three times in sterilized water, and then blotted on sterilized filter paper. About 1 g root segments were ground in 50 mL of sterile deionized water using a blender at high speed for 1 min. The homogenized root suspension was diluted 10 fold with sterile deionized water. 100 µL aliquots of diluted solutions were evenly spread on 4 plates containing Rose Bengal Medium using a sterile glass rod. There were 4 replicates and thus 16 plates for each treatment. All plates were incubated at 28°C in the dark for 4 days. Colonies of *C. parasiticum* were identified and counted on each plate to determine the CFUs per gram of roots for each treatment [18].

Another sand culture experiment was carried out for pathogen-related (*PR*) gene expression analysis. Except for the inoculation process, all the treatments were the same as above. Plants were grown in sand for thirty days without inoculation, and then inoculated with *C. parasiticum*. Spore suspensions of *C. parasiticum* were obtained from 14-day-old V8-juice media which were collected by adding 10 mL of sterile water to each Petri dish and rubbing the surface with a sterile L-shaped spreader. The suspension was subsequently filtered through 3-layers of cheesecloth. The spore concentration was determined using a hemacytometer and adjusted to 1×10^5^ spores per mL [18]. Thirty days after planting, plant stem base was infected with 20 mL *C. parasiticum* spore suspensions each pot. Three soybean roots from each pot were randomly harvested at 1 and 5 days after inoculation (DAI) to detect the expression pattern of eight related *PR* genes in response to inoculation using quantitative real time PCR (RT-PCR) described by Gao et al. [18]. Specific primer sequences and putative functions of tested plant *PR* genes were listed in the Table S1 in File S2. Simultaneously, the activities of polyphenol oxidase (PPO) and phenylalanine ammonia-lyase (PAL) were determined based on methods reported in Song et al. [21].

### Root exudates composition and *in vitro C. parasiticum* assays

A hydroponic experiment was carried out to collect root exudates in 2012. Soybean and/or maize seedlings were grown in half strength Hoagland nutrient solution with either of the two P additions (LP and HP) as described above. The cultivation modes included monoculture maize (MC) or soybean (MS), and intercropping of soybean and maize (ISC). There were six treatments (three cultivation modes by 2 P levels) with four replicates. Thirty days after planting, root exudates from each treatment were collected and processed, and a bioassay for colony diameter growth and sporulation was conducted as described in Gao et al. [18]. Roots were gently removed from pots and washed with deionized water. Cleaned roots were submerged in a plastic cup containing 500 mL of 0.5 µM CaCl_2_ to collect exudates for 6 hours. Root exudates were filtered through a 0.45 µm Millipore membrane and stored at −20 °C. During collection, each cup containing 3 plants was covered by a black plastic lid to avoid contamination and light [7], [22]. Pathogen growth diameter and sporulation was quantified by adding 2 mL root exudates to V8-juice medium before it solidified to yield a total volume of 20 mL per Petri dish. Plates were incubated at 28 °C in the dark. Colony diameter and sporulation of *C. parasiticum* were determined 5 and 14 days after incubation using ruler and hemocytometer [18]. Phenolic compounds in the root exudates were identified using an HPLC system (SPD-20A, Shimadzu, Tokyo) with gallic acid, p-coumaric acid, phthalic acid, vanillic acid, syringic acid, ferulic acid, salicylic acid and cinnamic acid included as standard phenolic compounds [22]. According to the above HPLC analyses results, the dominant phenolic acids, including ferulic, gallic, *p*-coumaric, cinnamic and salicylic acids (Sigma, USA), were subsequently applied exogenously at five concentrations, 0, 10, 20, 30 and 40 mg/L, in V8-juice media to test for allelopathic effects on growth and sporulation of *C. parasiticum* as described above.

### Data analysis

Data were statistically analyzed by Two-way ANOVA using Excel 2003 (Microsoft Corporation, 1985–2003) and SAS 9.1 (SAS Inc., Cary, NC, USA) for multiple comparisons.

## Results

### Reduction of soybean growth and yield caused by red crown rot in the field

Red crown rot significantly inhibited soybean growth, and subsequently reduced soybean yield in comparison to uninfected plants in field experiments (Figure B in File S1). Compared to healthy plants, infected soybean biomass in the monoculture treatment was reduced in 2009 by 23% and 27%, and in 2010 by 28% and 43% at LP and HP, respectively. Soybean yield was reduced in 2009 by 21% and 33%, and in 2010 by 17% and 44% at LP and HP, respectively. These observations illustrate the severe damage potential of red crown rot on soybean, particularly with high P supply.

### Disease severity of soybean red crown rot in the field

Intercropping significantly reduced disease incidence and severity index of soybean red crown rot, and high P slightly enhanced red crown rot in both monoculture and intercropping (Table 1). The planting distance of soybean and maize plants also significantly affected the disease severity as indicated by the lowest disease incidence and index occurring when soybean was grown in close proximity with maize (ISC2, 5 cm apart). Compared to monoculture soybean (MS), the disease incidence in ISC2 was reduced by 53% and 59% in 2009, and by 43% and 47% in 2010 at LP and HP, respectively. The disease index in ISC2 was decreased by 49% and 46% in 2009, and by 57% and 50% in 2010, at LP and HP, respectively (Table 1). This suggests that the occurrence and development of red crown rot on soybean growth can be alleviated by intercropping with maize, with particular attention paid to planting distance.

**Table 1:** Disease severity of soybean caused by *C. parasiticum* as affected by cultivation mode, P level and planting distance in the two years of field experiments on acid soils.

		Incidence (%)		Disease index	
		LP	HP	LP	HP
2009	MS	43±3.4Ab	62±5Aa	33±3Aa	40±3Aa
	ISC1	32±2Ba	39±4Ba	26±1Ab	32±2Ba
	ISC2	20±2Ca	26±2Ca	17±1Ba	22±3Ca
2010	MS	45±3Ab	58±4Aa	36±3Ab	48±2Aa
	ISC1	33±1Bb	41±2Ba	26±2Bb	36±2Ba
	ISC2	26±2Ca	31±1Ca	16±1Cb	24±2Ca

Note: Disease incidence and index were measured as described in Materials and Methods. HP: 80 kg P_2_O_5_ ha^−1^ added as calcium superphosphate, LP: no P fertilizer added. MS: soybean monoculture, ISC1: maize/soybean intercropping with 20 cm spacing; ISC2: maize/soybean intercropping with 5 cm spacing. All the data are the mean of four replicates ± SE. The same upper-case letter after numbers in the same column for the same trait in the same year indicates no significant difference among cultivation modes at 0.05 (*P*<0.05); The same lower-case letter after numbers in the same row for the same trait in the same year indicates no significant difference between two P levels at 0.05 (*P*<0.05).

### Mycorrhization, nodulation and root interactions in the field

**Figure 1:**
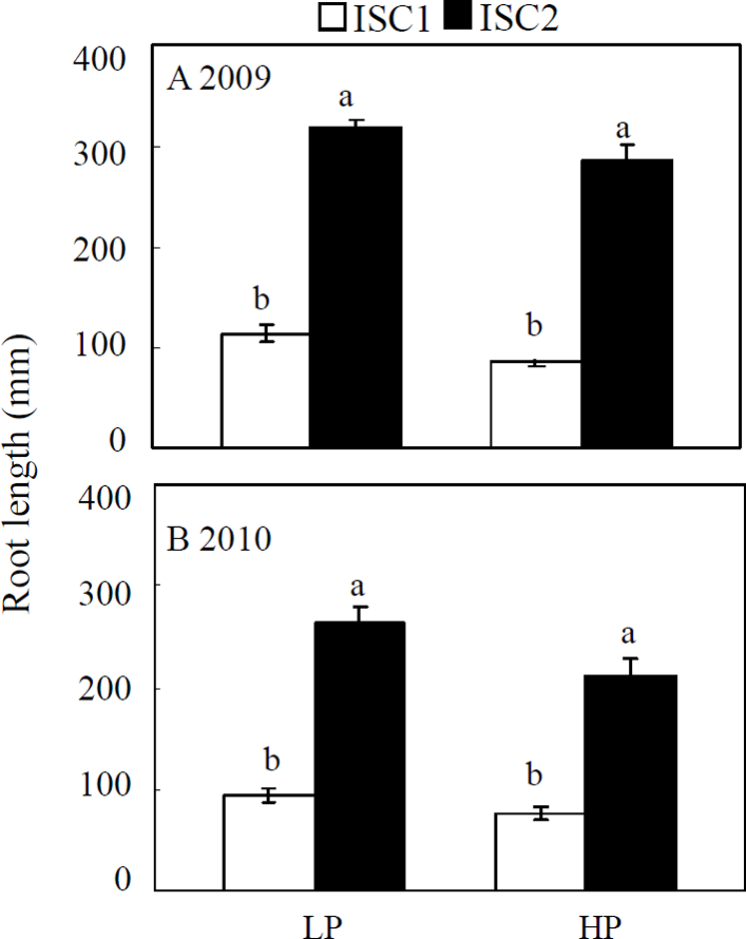
Root interactions between soybean and maize plants in the field. Root interactions were measured as the total root length in the upper 20: 80 kg P_2_O_5_ ha^−1^ added as calcium superphosphate, LP: no P fertilizer added. ISC1: maize/soybean intercropping with 20 cm spacing; ISC2: maize/soybean intercropping with 5 cm spacing. All the data are the mean of four replicates ± SE. F value from Two-way ANOVA: 8.35 for P treatment (*P*<0.05), 159.08 for cultivation mode (*P*<0.001), 1.99 for interaction (not significant). Bars with different letter(s) vary significantly among treatments as determined by Duncan's multiple range test (*P*<0.05).

Co-inoculation with AMF and rhizobia has been shown to alleviate soybean red crown rot [18]. However, in this study, the mycorrhization rate and nodule numbers were not affected by intercropping or planting distance (Table S2 in File S2), which indicates that neither mycorrhization nor nodulation are major contributors to the variation of soybean red crown rot observed in the field. In contrast, intermingled roots between the two different crop species were significantly affected by planting distance (Fig. 1). The total root length in the upper 20 cm soil layer of the 5 cm section centrally located between plants in ISC2 was greater in 2009 by 179% and 234% compared to ISC1 (20 cm apart), and in 2010 by 183% and 181% at LP and HP, respectively. Moreover, low P supply also affected root interactions. The root length in the upper, middle area at low P compared to high P increased by 33% and 24% in ISC1, and by 11% and 25% in ISC2 in 2009 and 2010, respectively (Fig. 1). This suggests that the intensity of intermingled roots between soybean and maize plants might be the major player in reducing the disease severity of soybean red crown rot by maize/soybean intercropping.

### Disease severity of soybean red crown rot in sand culture

**Figure 2:**
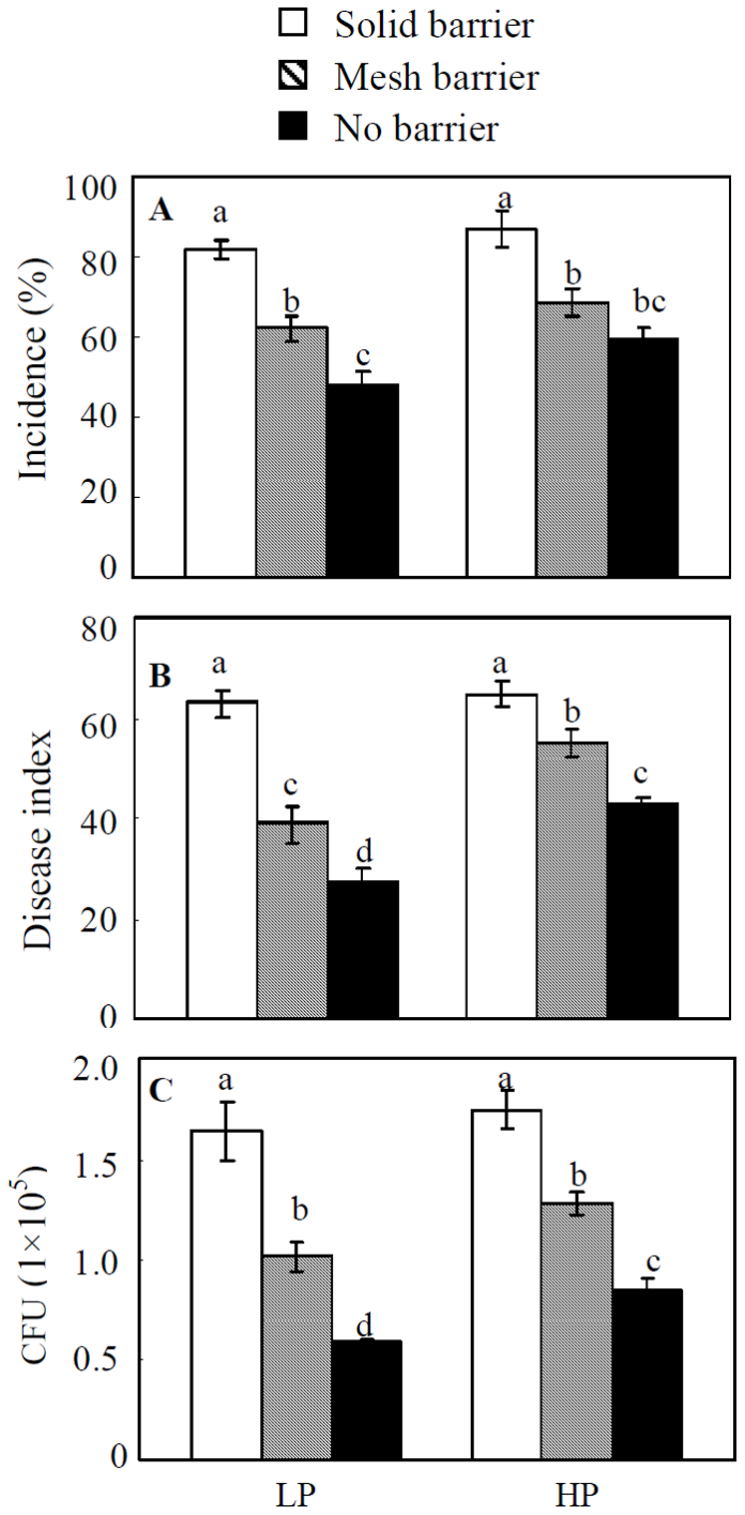
Disease severity of soybean red crown rot in sand culture. A, disease incidence, B, disease index, C, CFU. LP, 15 µM P; HP, 500 µM P. All of the data are the means of four replicates ±SE. Bars with different letter(s) vary significantly among the different inoculation treatments as determined by Duncan's multiple range test (*P*<0.05).

The disease severity of soybean red crown rot was dramatically decreased with increasing root interactions in sand culture. Compared to the solid barrier treatment, both mesh and no barrier treatments had significantly lower disease incidences and indices, and fewer pathogen CFUs at both P levels (Fig. 2, Figure C in File S1). When soybean and maize roots completely interacted with each other (no barrier), the lowest disease incidences and indices and fewest pathogen CFUs were observed. An intermediate level of disease incidence was observed in plants grown with the mesh barrier treatment, and the highest level of disease was seen in plants separated by solid barriers. This is consistent with field results that an increase in root interactions reduced the occurrence of soybean red crown rot.

### Expressions of important *PR* genes and activities of corresponding enzymes in sand culture

**Figure 3:**
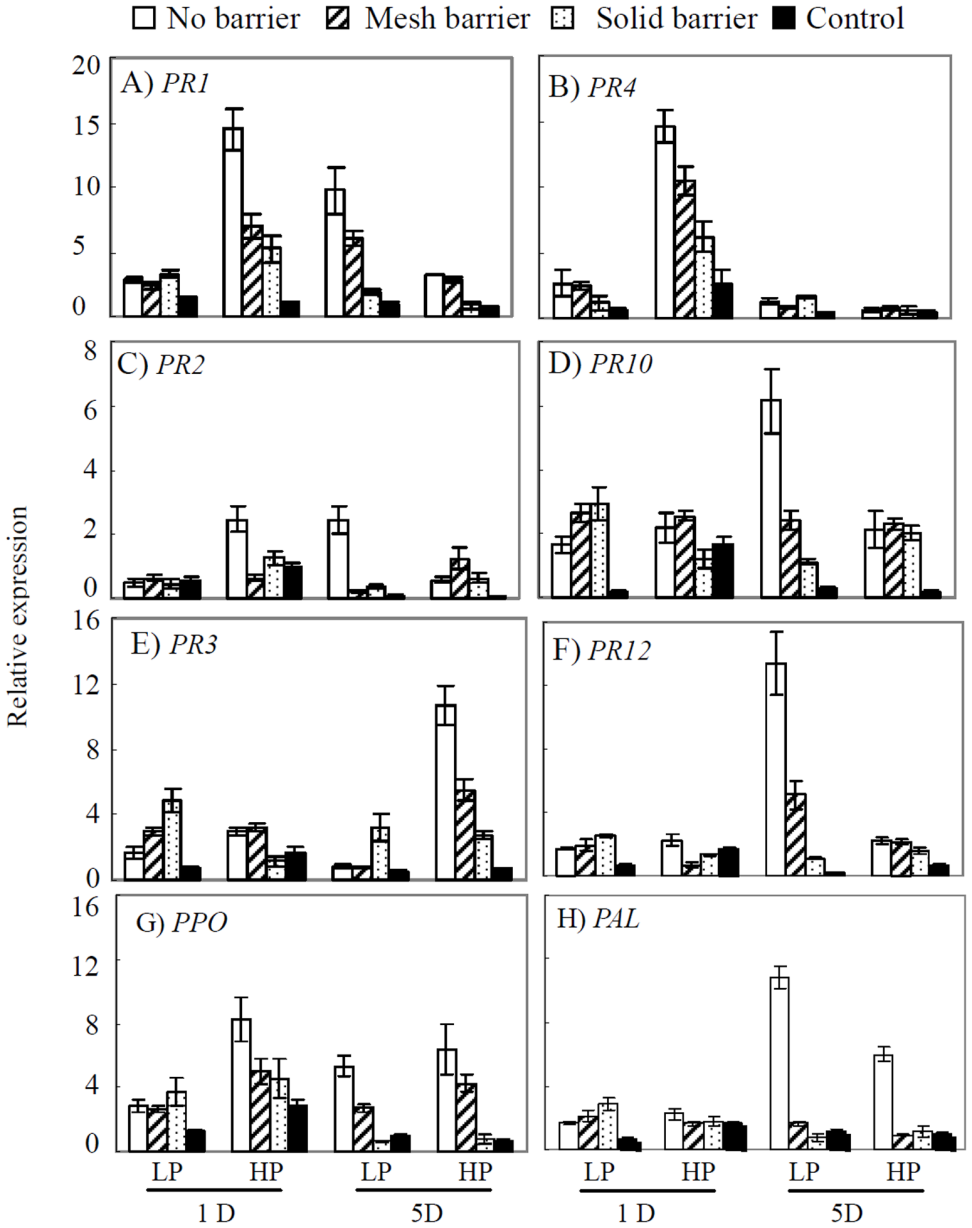
Expression of eight defense-related (*PR*) genes in soybean roots. LP: 15 µM P; HP: 500 µM P. Soybean roots were inoculated with *C. parasiticum* (see Materials and Methods for details). Solid barrier: eliminate root interactions and exudates movement between the roots of the two plant species; mesh barrier: prevent root intermingling of two species while permitting root exudates exchange; no barrier: allow roots and exudates to completely interact. Each bar represents the mean of three replicates ± SE.

The results displayed in Figure 3 showed that transcript abundances of all tested *PR* genes in soybean roots were induced to a high level by inoculation with *C. parasiticum*, and this induction was enhanced by increasing root interactions. Except *PR4* on day 5 and *PR3*, *PR10*, *PR12* and *PAL* on day 1, the relative expression values of most tested *PR* genes were highest in the no barrier treatment, followed by the mesh barrier treatment. For example, *PPO* transcription abundance increased by 7.3- and 7.4- fold, and *PAL* by 12.9- and 4.1- fold in LP and HP, respectively, at 5 DAI in a comparison of the no barrier treatment with the solid barrier treatment (Fig. 3G and H). Furthermore, the enzyme activities of PPO and PAL in soybean roots were significantly enhanced by root interactions with maize (Figure D in File S1). PPO activity increased in the no barrier treatment compared to the solid barrier treatment at 1 DAI by 114% and 99%, and in the mesh treatment by 66% and 43% in LP and HP, respectively. Also, PAL activity increased in the no barrier treatment compared to the solid barrier treatment at 1 DAI by 65% and 227%, and in the mesh treatment by 39% and 105% in LP and HP, respectively.

### *In vitro C. parasiticum* assays using root exudates

**Figure 4:**
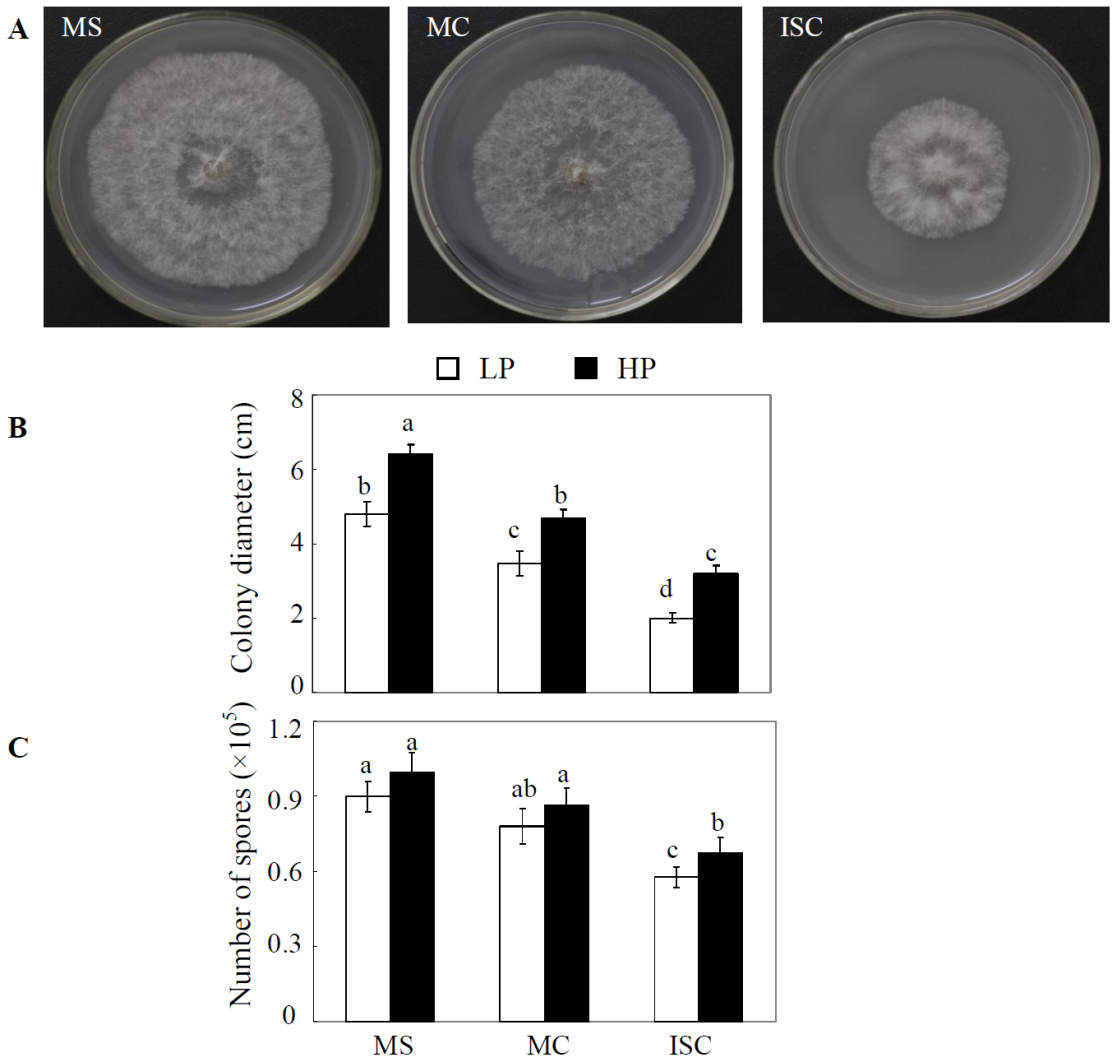
*C. parasiticum* growth as affected by root exudates. A, growth performance, B, colony diameter, C, sporulation. LP, 15 µM P; HP, 500 µM P. MS: soybean monoculture; MC: maize monoculture; ISC: maize/soybean intercropping. F value from Two-way ANOVA: colony diameter, 685.62 for P treatment (*P*<0.001), 1119.75 for cultivation mode (*P*<0.001), 12.73 for interaction (*P*<0.001); sporulation, 38.33 for P treatment (*P*<0.001), 116.49 for cultivation mode (*P*<0.001), 1.02 for interaction (not significant). Each bar represents the mean of four replicates ± SE. Bars with different letter(s) vary significantly among treatments as determined by Duncan's multiple range test (*P*<0.05).

In comparison with soybean monoculture (MS), the addition of root exudates from the maize/soybean intercropping (ISC) or maize monoculture (MC) dramatically inhibited pathogen growth (Fig. 4). At LP, colony diameter of *C. parasiticum* decreased by 54% and 38%, and sporulation declined by 37% and 27% when growth in MS exudates was compared to that in ISC or MC exudates, respectively. Furthermore, P supply also affected pathogen growth as indicated by the observed 34%, 31% and 49% increases in colony diameter in HP compared to LP when adding the root exudates from MS, MC and ISC, respectively. This suggests that maize roots can be triggered to secrete antagonistic substances against soybean pathogen growth in the intercropping system, which might be enhanced under low P supply.

### HPLC analysis of root exudates

We investigated the composition of root exudates using HPLC analysis, and found that the composition of root exudates varied significantly among the two crop species and the different cultivation modes (i.e., monoculture of soybean or maize, intercropping with soybean and maize). Roots of maize plants released greater amounts of phenolic acids than those of soybean (Figure E in File S1). We further analyzed the presence and levels of 8 common phenolic acids in the root exudates using HPLC with chemical standards. This analysis identified 5 phenolic acids, including ferulic, gallic, *p*-coumaric, cinnamic and salicylic acids, in the root exudates from MC or ISC, but only gallic acid was detected in soybean monoculture root exudates (Table 2). The concentration of the phenolic acids other than gallic acid in root exudates increased when maize was intercropped with soybean. For example, cinnamic acid concentration in exudates from the ISC treatment compared to the MC treatment increased by 20% and 43% at LP and HP, respectively. Phosphorus supply also influenced the concentrations of most phenolic acids. Ferulic, *p*-coumaric and cinnamic acids in root exudates of the ISC grown plants exhibited 68%, 58% and 22% increases at LP compared to HP.

**Table 2:** Concentrations of phenolic acids (µg·pot^−1^) in root exudates from different treatments at the flowering stage as detected by HPLC.

Treatment		Ferulic acid	Gallic acid	*P*-coumaric acid	Cinnamic acid	Salicylic acid
	MS	-	6.53±1.59c	-	-	-
LP	MC	12.78±2.34b	29.08±0.80a	13.06±1.91b	20.98±2.51b	7.51±1.41a
	ISC	22.13±3.24a	14.72±2.84b	20.33±2.78a	25.11±2.52a	7.88±2.72a
	MS	-	11.12±2.88b	-	-	-
HP	MC	13.72±1.05a	13.09±1.58b	8.51±0.26a	14.41±2.86b	11.00±2.21a
	ISC	13.18±1.73a	21.65±2.99a	12.86±2.28a	20.59±2.78a	9.32±0.47a

Note: LP, 15 µM P; HP, 500 µM P. MS, soybean monoculture; MC, maize monoculture; ISC, maize/soybean intercropping. -, not detectable. All the data are the mean of four replicates ± SE. Data with the same letter represent no significant differences among the different cultivation modes at the same P level as determined by Duncan's multiple range test or a *t*-test (*P*<0.05).

### Effects of phenolic acids on *C. parasiticum* growth

**Figure 5:**
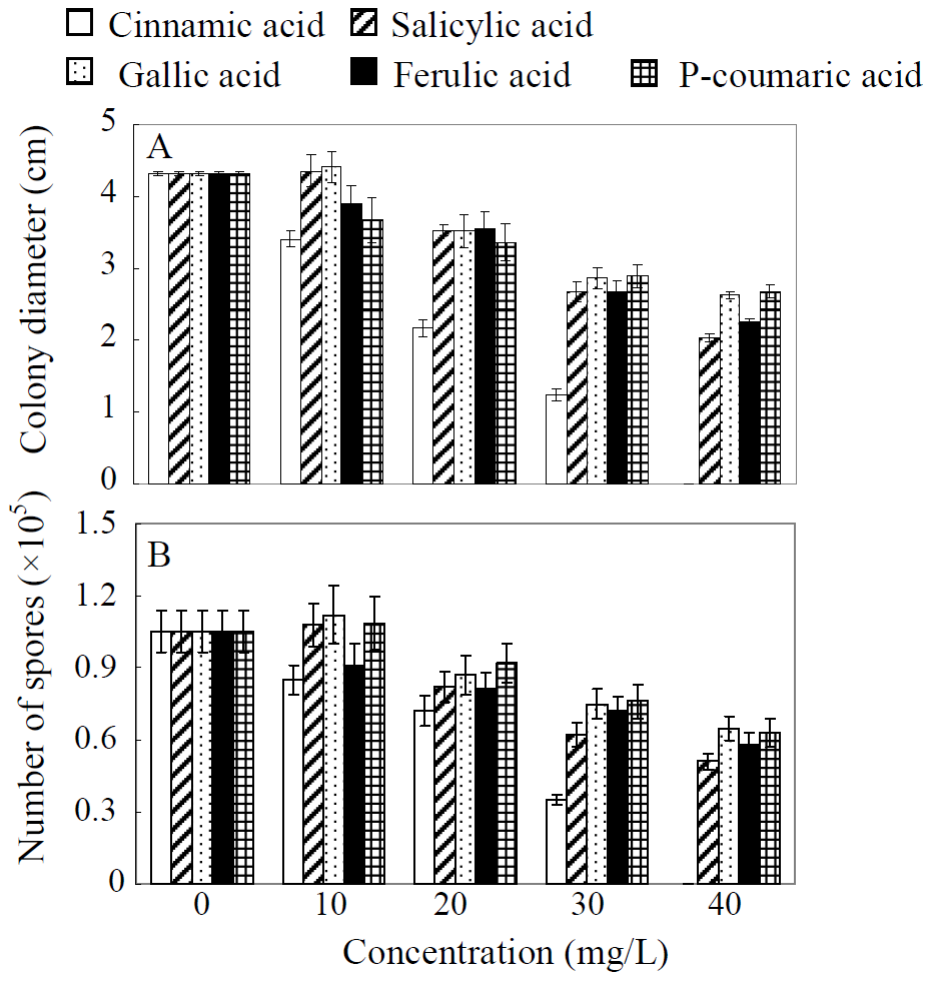
Effect of 5 different phenolic acids on the growth and sporulation of *C. parasiticum*. A, colony diameter, B, number of spores. Each bar represents the mean of four replicates ± SE.

To further investigate the effects of the above five phenolic acids on *C. parasiticum* growth and sporulation, we conducted an *in vitro C. parasiticum* assay using individual phenolic acids. The results showed that when the concentration reached 20 mg/L, all of the tested phenolic acids inhibited the growth and sporulation of *C. parasiticum* (Fig. 5). At lower concentrations, only cinnamic and ferulic acids inhibited *C. parasiticum* growth, while, lower concentration gallic and salicylic acids slightly stimulated *C. parasiticum* growth. Furthermore, the inhibitory effects of cinnamic acid on *C. parasiticum* growth increased with increasing concentration, and even completely stopped *C. parasiticum* growth when the concentration reached 40 mg/L. These results suggest that cinnamic acid is the most effective phenolic acid in suppressing *C. parasiticum* growth and development.

## Discussion

It has been well documented that increasing crop species diversity can yield benefits in plant disease control [2], [3]. However, most studies have focused on above-ground plant functions and performances. Few plant disease studies pay attention to the root, the main organ of plants to sense environmental attacks and related signals in the soil. To the best of our knowledge, this is the first report on interspecies signaling between root systems that results in improved control of soil-borne disease, with evidence coming both from field trials and greenhouse-laboratory experiments at both physiological and molecular levels.

Intercropping is the intermingled growth of at least two crops grown together in the same field, which is the most popular cultivation model for increasing crop genetic diversity [23]. It has been widely noted that intercropping systems can reduce disease damage [4], [15], [24], probably due to fewer attacks by disease organisms compared to when crops are grown in monoculture [2], [24]. In the current study, based on two years of field experiments where soybean red crown rot was severe in monoculture, we found that intercropping soybean with maize significantly reduced disease severity of soybean red crown rot (Table 1). This was further confirmed in sand culture with different root barrier treatments. Most significantly, when root interactions were completely blocked, severe red crown rot still occurred in soybean plants, even when intercropped with maize (Fig. 2). This indicates that above-ground interactions in the maize/soybean intercropping system do not contribute to control of soybean red crown rot. In contrast, the least severe red crown rot was recorded when the roots freely interacted with each other in the no barrier treatment, indicating that interactions among roots in intercropping is important in control of this soil-borne disease. Moreover, we also found that in the field, the reduction of soybean red crown rot was obviously enhanced with decreasing planting distance between soybean and maize plants, which increased interaction intensity between the two different crop species (Table 1 and Fig. 1). This finding suggests that besides genetic diversity, the intensity of root interaction is also important for control of soybean red crown rot in the maize/soybean intercropping system. This point is also supported by reports that in order to effectively control disease spread, roots of mixed crops need to be sufficiently intermingled [24], [25].

Root exudates have been recognized to play vital roles in the rhizosphere [26]–[28] in large part due to continuous production and secretion of allelochemical compounds into the rhizosphere [29], [30]. There are many reports attributing defense against phytopathogens to root exudates released by neighboring non-host plants [6]–[7], [31]. In the present sand culture experiment, the mesh barrier treatment had less effects on disease control than the no barrier treatment, yet still significantly reducing the severity of soybean red crown rot (Fig. 2), implying that root exudates alone can inhibit soybean red crown rot. This was supported by *in vitro* assays where the significant growth inhibition of *C. parasiticum* was observed when root exudates were applied. Root exudates collected from intercropped soybean and maize inhibited *C. parasiticum* growth more effectively than from maize alone (Fig. 4), suggesting that intercropped maize releases more allelopathic compounds than in monoculture.

Phenolic acids have been well established as major rhizosphere allelochemicals that suppress phytopathogens [22], [32], and they are detectable in root exudates by HPLC [7], [29]. In order to identify the specific compounds inhibitory to *C. parasiticum* growth from the maize/soybean intercropping system, the 8 most common phenolic acids in root exudates were initially tested, with 4 phenolic acids specifically released by maize roots (Figure E in File S1). Among these 4 compounds, release of cinnamic acid was significantly enhanced by maize/soybean intercropping (Table 2), and this compound dramatically constrained *C. parasiticum* growth *in vitro* (Fig. 5). Cinnamic acid has been demonstrated to inhibit phytopathogen *Fusarium oxysporum* f.sp. *niveum in vitro* [7], [22]. Therefore, we speculate that cinnamic acid is the primary player of interest in the tested root exudates, and it is directly responsible for the protection of soybean roots from the attack of *C. parasiticum* in the maize/soybean intercropping system. Since plant roots can exude a broad range of compounds, a more thorough search for more efficient phytotoxins from soybean and/or maize needs to be carried out in the future.

The *PR* related genes included pathogenesis-related, antimicrobial protein genes *PR1* (PR1a precursor), *PR2* (β 1–3 endoglucanase), *PR3* (chitinase class I), *PR4* (wound-induced protein), *PR10* (ribonuclease-like protein), *PR12* (defending-like protein), *PPO* (polyphenol oxidase) and *PAL* (phenylalanine ammonia-lyase) that are involved in phytoalexin biosynthesis [33], [34]. Here we shown that the defense responses elicited by inoculation of pathogen and intercropping with maize. In legumes, many genes encoding PR proteins have been shown to be upregulated in plants following inoculation with several pathogens [33]. The upregulation of defense-related genes, such as *PR1* and *PR2* was associated with lesion limitation in soybean seedling roots inoculated with *P. sojae* and to the high level of partial resistance of cultivar Conrad [33]. Defense-related genes are upregulated in both compatible and incompatible interactions, but more rapidly and/or to higher levels in plant varieties with *PR* gene resistance [34]. In addition to producing allelopathic compounds directly, intercropped plants can promote specific plant defense reactions in neighboring plants to protect against phytopathogens [2], [31]. Consistent with this, the results herein show that direct interactions with maize roots significantly induce the expression of important soybean *PR* genes and increase the activities of corresponding PR enzymes in soybean roots (Fig. 3, Figure C in File S1). This elicitation, via root exudates or signal compounds for specific plant defense reactions can predispose soybean plants to an early response to attack by root pathogens [24], [31]. Infecting soybean roots after pre-treatment with maize enhances PR gene expression and enzyme activity, and therefore, might improve soybean's capability to defend against disease invasion. Furthermore, salicylic acid is a reported enhancer of plant defense pathways [35]. In this study, more salicylic acid was measured in root exudates from the maize/soybean intercropping system than in monoculture, which suggests that salicylic acid or a related component might also contribute to disease control through promotion of defense pathways.

Although adding additional P fertilizer in field and sand culture experiments increased plant growth, it also promoted disease incidence and index of red crown rot (Table 1 and Fig. 2). Likewise, the application of P has been shown to increase the severity of diseases in many plant species [18], [36]. Here, we demonstrate that higher P addition is associated with more severe soybean red crown rot (Table 1 and Fig. 2). Furthermore, high P induces colony *C. parasiticum* growth and proliferation *in vitro* (Fig. 4). Plant roots and phytopathogens most likely compete directly for resources (e.g. P) in the rhizosphere, and therefore low P supply could inhibit phytopathogen growth, especially if the roots more effectively acquire the soil P. Consequently, optimal fertilizer application should be carefully considered, especially in acid soils where P is limiting.

To the best of our knowledge, this study is the first report to demonstrate that intercropping with maize can promote disease resistance in soybean to red crown rot in a root interaction dependent manner. Direct root interactions of soybean with maize may inhibit pathogen growth and reproduction, while enhancing expression of *PR* genes and the activities of corresponding enzymes in host plants. This study suggests that soybean and maize intercropping is an effective tool to sustainably control soil-borne disease and should be incorporated in agricultural management practices.

## Supporting Information

File S1 **Supporting figures.** **Figure A.** Planting arrangement of diagram and actual pictures in monoculture or intercropping of soybean and maize. **A**, Planting arrangement in the field. **B**, Three root barriers were set-up in sand culture, either with a solid barrier (left) to eliminate root interactions and exudates movement, a nylon mesh barrier (middle) to prevent root intermingling of two species while permitting root exudates exchange, and no barrier (right) to allow roots and exudates to completely interact. **Figure B.** Soybean growth and grain yield as affected by red crown rot. HP: 80 kg P_2_O_5_ ha^−1^ added as calcium superphosphate, LP: no P fertilizer added. The reduction of growth parameters were calculated as follows: reduction (%)  =  (yield/biomass of healthy plants- yield/biomass of infected plants) yield/biomass of healthy plants ×100. Each bar represents the mean of four replicates ± SE. **Figure C.** Disease severity of soybean red crown rot in sand culture (Second round). A, disease incidence, B, disease index, C, CFU. LP, 15 µM P; HP, 500 µM P. All of the data are the means of four replicates ±SE. Bars with different letter(s) vary significantly among treatments as determined by Duncan's multiple range test (*P*<0.05). **Figure D.** Activities of the enzyme PPO (A) and PAL (B) in soybean roots. LP, 15 µM P; HP, 500 µM P. Except for control, all the roots were inoculated with *C. parasiticum* (see Materials and Methods for details). The solid barrier eliminated root contact and exudates movement, the nylon mesh (30 µm) barrier prevented root intermingling for the two species while permitting root exudates exchange, and no root barrier permitted roots and exudates to completely interact. Each bar represents the mean of three replicates ± SE. **Figure E.** HPLC scan of soybean and/or maize root exudates. **(a and b**) HPLC scan of soybean root exudates, (**c and d**) HPLC scan of maize root exudates, (**e and f**) HPLC scan of the root exudates from maize/soybean intercropping, (**g**) HPLC analysis of eight standard phenolic acids. LP, 15 µM P (a, c and e); HP, 500 µM P (b, d and f). 1, Gallic acid; 2, *P*-coumaric; 3, Phthalic acid; 4, Vanillic acid; 5, Syringic acid; 6, Ferulic acid; 7, Salicylic acid; 8, Cinnamic acid.
(PDF)

File S2 **Supporting tables.** **Table S1.** Real time PCR primers designed for this study. **Table S2.** AMF colonization rate and nodule number of soybean in the field.
(DOC)

## References

[1] Ratnadass A, Fernandes P, Avelino J, Habib R (2012) Plant species diversity for sustainable management of crop pests and diseases in agroecosystems: a review. Agronomy for Sustainable Development 32: 273–303.

[2] Zhu Y, Chen H, Fan J, Wang Y, Li Y, et al (2000) Genetic diversity and disease control in rice. Nature 406: 718–722. 10963595 10.1038/35021046

[3] Browning JA, Frey KJ (1969) Multiline cultivars as a means of disease control. Annual Review of Phytopathology 7: 355–382.

[4] Smedegaard-Petersen V (1985) The limiting effect of disease resistance on yield. Annual Review of Phytopathology 23: 475–490.

[5] Yu JQ (1999) Allelopathic suppression of Pseudomonas solanacearum infection of tomato (*Lycopersicon esculentum*) in a tomato-chinese chive (*Allium tuberosum*) intercropping system. Journal of Chemical Ecology 25: 2409–2417.

[6] Ren LX, Su SM, Yang XM, Xu YC, Huang QW, et al (2008) Intercropping with aerobic rice suppressed Fusaium wilt in watermelon. Soil Biology Biochemistry 40: 834–844.

[7] Hao WY, Ren LX, Ran W, Shen QR (2010) Allelopathic effects of root exudates from watermelon and rice plants on *Fusarium oxysporum* f.sp. *niveum* . Plant and Soil 336: 485–497.

[8] Gómez RO, Zavaleta M, González H, Livera M, Cárdenas S (2003) Allelopathy and microclimatic modification of intercropping with marigold on tomato early blight disease development. Field Crops Research 83: 27–34.

[9] Bell DK, Sobers EK (1966) A peg, pod and root necrosis of peanuts caused by a species of *Calonectria* . Phytopathology 56: 1361–1364.

[10] Kuruppu P, Schneider R, Russin J (2004) Factors affecting soybean root colonization by *Calonectria ilicicola* and development of red crown rot following delayed planting. Plant Disease 88: 613–619. 10.1094/PDIS.2004.88.6.613 30812580

[11] Guan M, Pan R, Gao X, Xu D, Deng Q, et al (2010) First report of red crown rot caused by *Cylindrocladium parasiticum* on soybean in Guangdong, Southern China. Plant Disease 94: 485. 10.1094/PDIS-94-4-0485B 30754497

[12] Li L, Zhang F, Li X, Peter C, Sun J, et al (2003) Interspecific facilitation of nutrient uptake by intercropped maize and faba bean. Nutrient Cyclling in Agroecosystems 65: 61–71.

[13] He Y, Ding N, Shi JC, Wu M, Liao H, et al (2013) Profiling of microbial PLFAs: Implications for interspecific interactions due to intercropping which increase phosphorus uptake in phosphorus limited acidic soils. Soil Biology Biochemistry 57: 625–634.

[14] Ae N, Arihara J, Okada K, Yoshihara T, Johansen C (1990) Phosphorus uptake by pigeon pea and its role in cropping systems of Indian subcontinent. Science 248: 477–480. 17815599 10.1126/science.248.4954.477

[15] Li C, He X, Zhu S, Zhou H, Wang Y, et al (2009) Crop diversity for yield increase. PLoS ONE 4: e8049. 19956624 10.1371/journal.pone.0008049 PMC2778130

[16] Fang SQ, Gao X, Deng Y, Chen XP, Liao H (2011) Crop root behavior coordinates phosphorus status and neighbors: from field studies to three-dimensional in situ reconstruction of root system architecture. Plant Physiology 155: 1277–1285. 21224339 10.1104/pp.110.167304 PMC3046585

[17] Fang S, Clark RT, Zheng Y, Iyer-Pascuzzia AS, Weitzf JS, et al (2013) Genotypic recognition and spatial responses by rice roots. Proceedings of the National Academy of Sciences, USA 110: 2670–2675. 10.1073/pnas.1222821110 PMC3574932 23362379

[18] Gao X, Lu X, Wu M, Zhang HY, Pan RQ, et al (2012) Co-inoculation with rhizobia and AMF inhibited soybean red crown rot: from field study to plant defense-related gene expression analysis. PLoS ONE 7: e33977. 22442737 10.1371/journal.pone.0033977 PMC3307780

[19] Wang XR, Pan Q, Chen FX, Yan XL, Liao H (2011) Effects of co-inoculation with arbuscular mycorrhizal fungi and rhizobia on soybean growth as related to root architecture and availability of N and P. Mycorrhiza 21: 173–181. 20544230 10.1007/s00572-010-0319-1

[20] Liao H, Wan HY, Shaff J, Wang XR, Yan XL, et al (2006) Phosphorus and aluminum interactions in soybean in relation to aluminum tolerance. Exudation of specific organic acids form different regions of the intact root system. Plant Physiology 141: 674–684. 16648222 10.1104/pp.105.076497 PMC1475464

[21] Song YY, Zeng RS, Xu JF, Li J, Shen X, et al (2010) Interplant communication of tomato plants through underground common mycorrhizal networks. PLoS ONE 5: e13324. 20967206 10.1371/journal.pone.0013324 PMC2954164

[22] Ling N, Huang QW, Guo SW, Shen QR (2011) Paenibacillus polymyxa SQR-21 systemically affects root exudates of watermelon to decrease the conidial germination of *Fusarium oxysporum* f.sp. *niveum* . Plant and Soil 341: 485–493.

[23] Li L, Li SM, Sun JH, Zhou LL, Bao XG, et al (2007) Diversity enhances agricultural productivity via rhizosphere phosphorus facilitation on phosphorus-deficient soils. Proceedings of the National Academy of Sciences, USA 104: 11192–11196. 10.1073/pnas.0704591104 PMC1899187 17592130

[24] Trenbath BR (1993) Intercropping for the management of pests and diseases. Field Crops Research 34: 381–405.

[25] Meynard JM, Dore T, Lucas P (2003) Agronomic approach: cropping systems and plant disease. Comptes Rendus Biologies 326: 37–46. 12741180 10.1016/s1631-0691(03)00006-4

[26] Hoffland E, Findenegg G, Nelemans J, van den Boogaard R (1992) Biosynthesis and root exudation of citric and malic acids in phosphate-starved rape plants. New Phytologist 122: 675–680.

[27] Bardgett RD, Denton CS, Cook R (1999) Below-ground herbivory promotes soil nutrient transfer and root growth in grassland. Ecology Letters 2: 357–360.

[28] Bais HP, Prithiviraj B, Jha AK, Ausubel FM, Vivanco JM (2005) Mediation of pathogen resistance by exudation of antimicrobials from roots. Nature 434: 217–221. 15759001 10.1038/nature03356

[29] Bais HP, Vepachedu R, Gilroy S, Callaway RM, Vivanco JM (2003) Allelopathy and exotic plant invasion: from molecules and genes to species interactions. Science 301: 1377–1380. 12958360 10.1126/science.1083245

[30] Weir TL, Park SW, Vivanco JM (2004) Biochemical and physiological mechanisms mediated by allelochemicals. Current Opinion in Plant Biology 7: 472–479. 15231272 10.1016/j.pbi.2004.05.007

[31] Bais HP, Weir TL, Perry LG, Gilroy S, Vivanco JM (2006) The role of root exudates in rhizosphere interactions with plants and other organisms. Annual Review of Plant Biology 57: 233–266. 10.1146/annurev.arplant.57.032905.105159 16669762

[32] Wu HS, Liu DY, Ling N, Bao W, Ying RR, et al (2009) Influence of root exudates of wantermelon on *Fusarium oxysporum* f. sp. *niveum* . Soil Biology Biochemistry 73: 1150–1156.

[33] Robert GU, Martha ER (2010) Defense-related gene expression in soybean leaves and seeds inoculated with *Cercospora kikuchii* and *Diaporthe phaseolorum* var. *Meridionalis* . Physiological and Molecular Plant Pathology 75: 64–70.

[34] Van Loon LC, Rep M, Pirterse CMJ (2006) Significance of inducible defense-related proteins in infected plants. Annual Review of Phytopathology 44: 135–162. 10.1146/annurev.phyto.44.070505.143425 16602946

[35] Wees S, Swart E, Pelt J, Loon L, Pieterse C (2000) Enhancement of induced disease resistance by simultaneous activation of salicylate- and jasmonate-dependent defense pathways in Arabidopsis thaliana. Proceedings of the National Academy of Sciences, USA 97: 8711–8716. 10.1073/pnas.130425197 PMC27013 10890883

[36] Dordas C (2008) Role of nutrients in controlling plant disease in sustainable agriculture: a review. Agronomy for Sustainable Development 28: 33–46.

