# Supplementary figures and images for: Root Interactions in a Maize/Soybean Intercropping System Control Soybean Soil-Borne Disease, Red Crown Rot
Source: PLoS One. 2014 May 8;9(5):e95031. doi: 10.1371/journal.pone.0095031 (PMC4014482; doi:10.1371/journal.pone.0095031)

Figure A

A

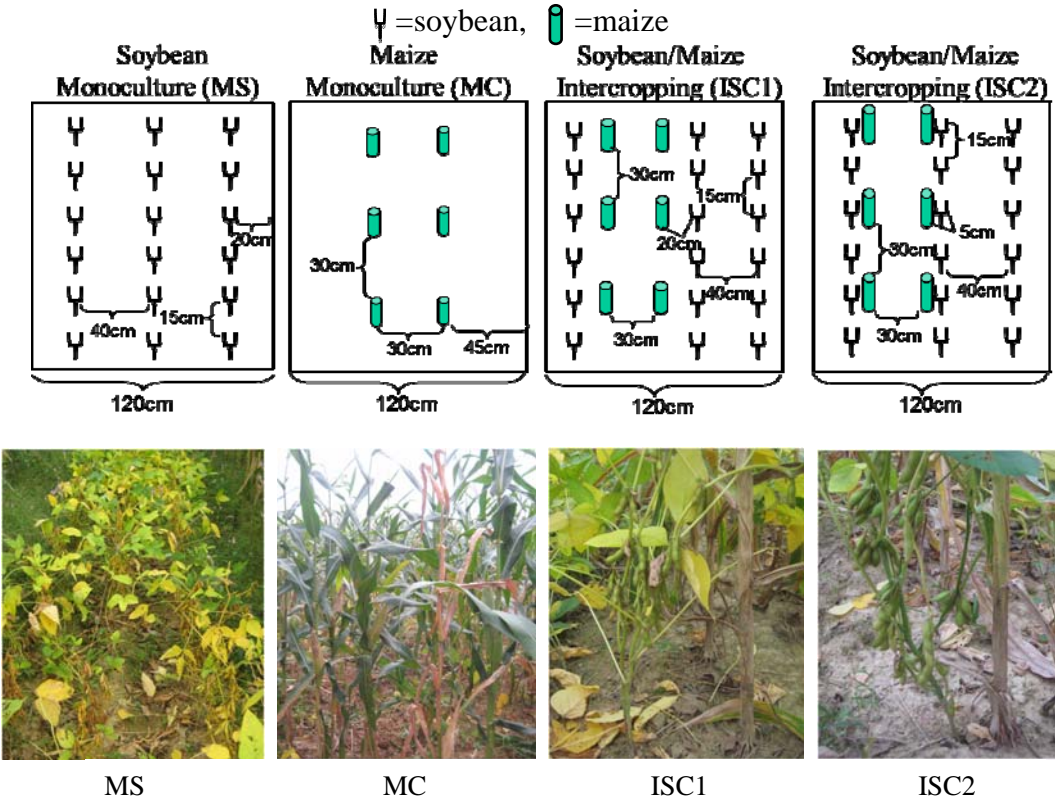

B

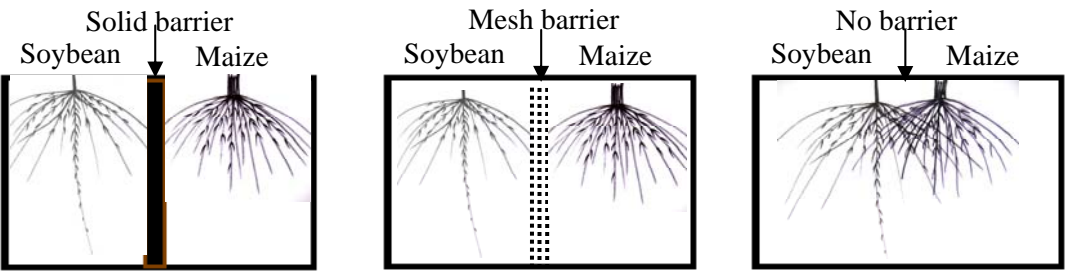

**Figure B**

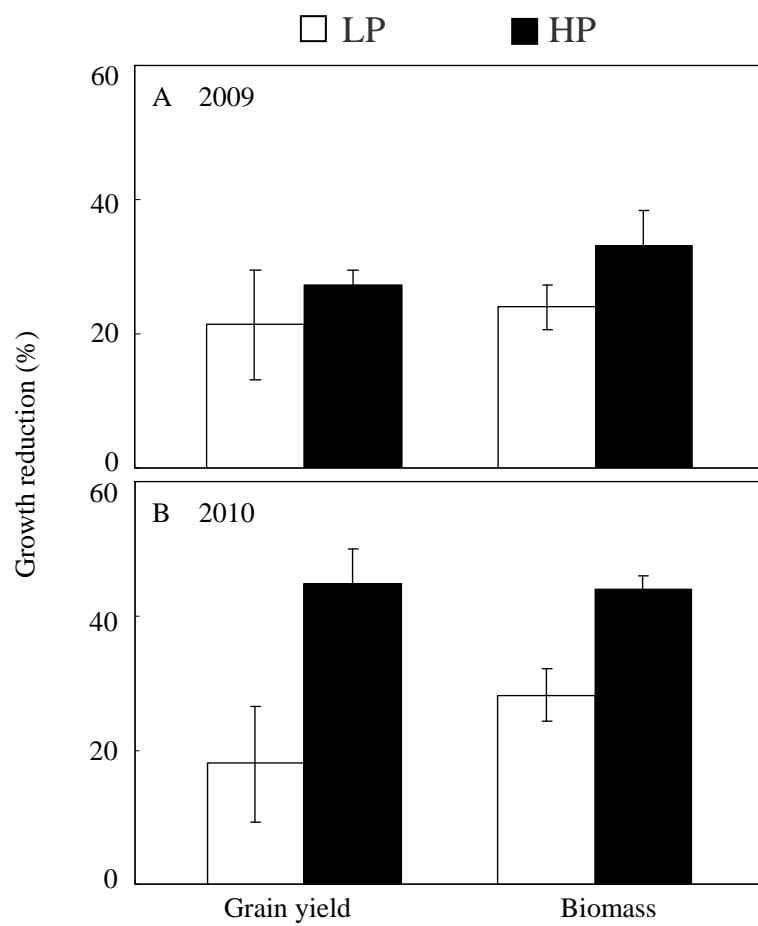

**Figure C**

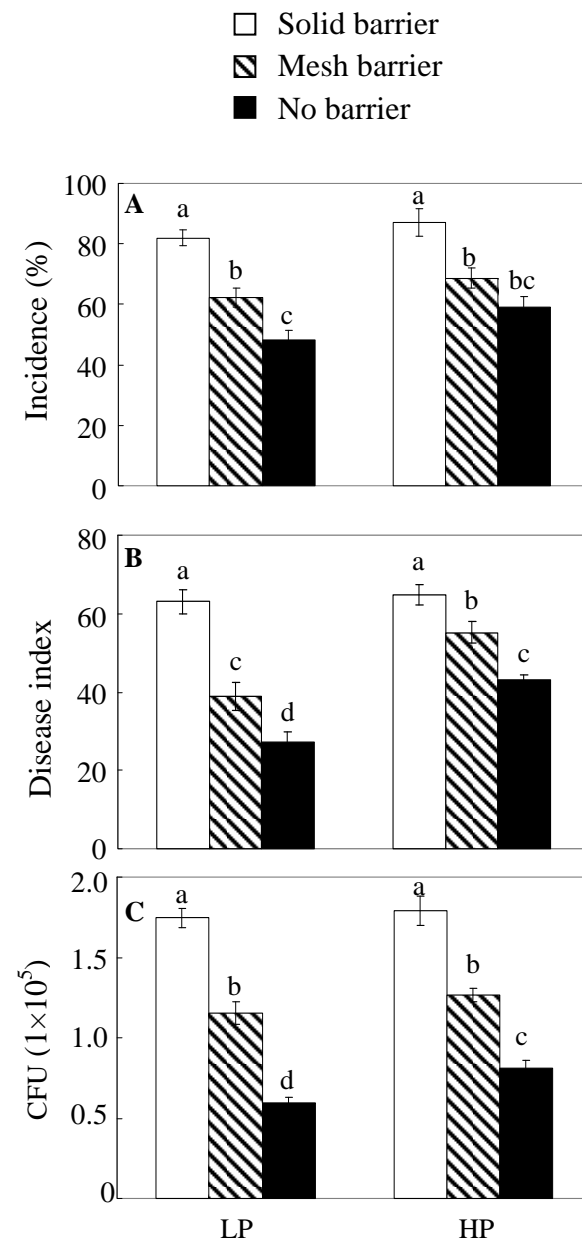

**Figure D**

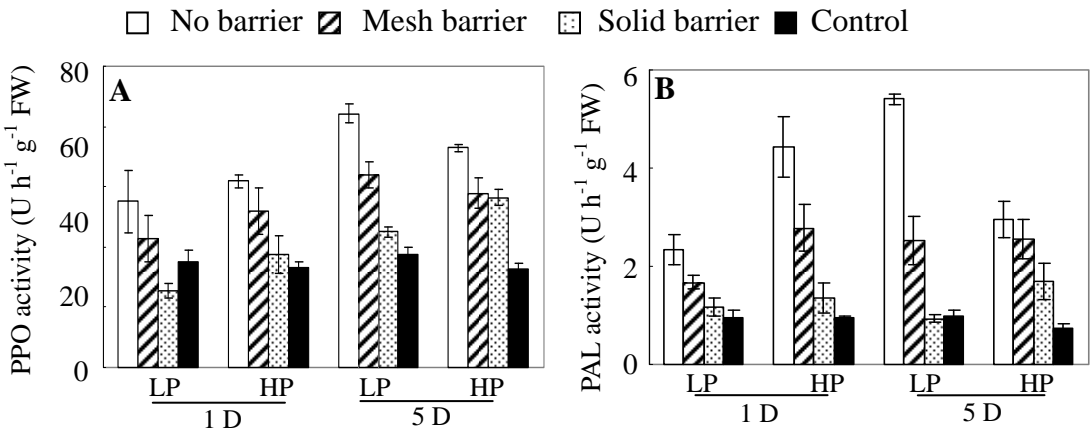

**Figure E**

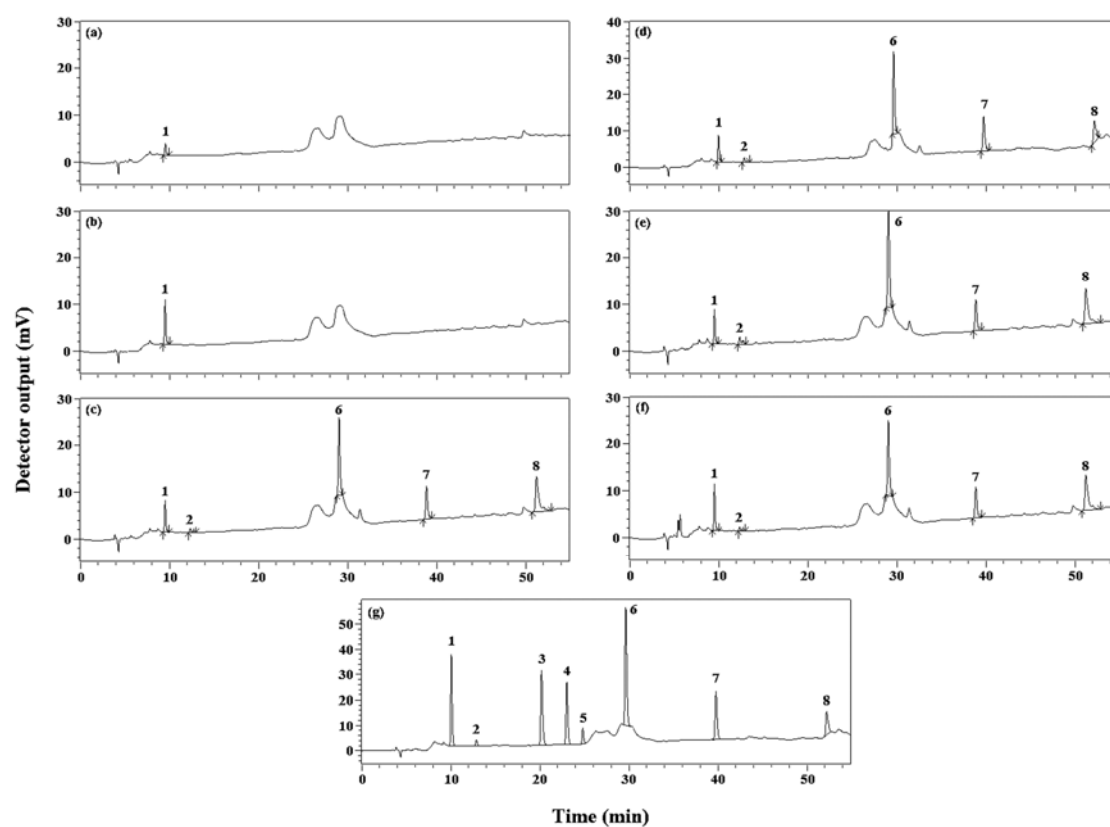

Supplement: File S1 — Supporting figures. Figure A. Planting arrangement of diagram and actual pictures in monoculture or intercropping of soybean and maize. A, Planting arrangement in the field. B, Three root barriers were set-up in sand culture, either with a solid barrier (left) to eliminate root interactions and exudates movement, a nylon mesh barrier (middle) to prevent root intermingling of two species while permitting root exudates exchange, and no barrier (right) to allow roots and exudates to completely interact. Figure B. Soybean growth and grain yield as affected by red crown rot. HP: 80 kg P2O5 ha−1 added as calcium superphosphate, LP: no P fertilizer added. The reduction of growth parameters were calculated as follows: reduction (%) = (yield/biomass of healthy plants- yield/biomass of infected plants) yield/biomass of healthy plants ×100. Each bar represents the mean of four replicates ± SE. Figure C. Disease severity of soybean red crown rot in sand culture (Second round). A, disease incidence, B, disease index, C, CFU. LP, 15 µM P; HP, 500 µM P. All of the data are the means of four replicates ±SE. Bars with different letter(s) vary significantly among treatments as determined by Duncan's multiple range test (P<0.05). Figure D. Activities of the enzyme PPO (A) and PAL (B) in soybean roots. LP, 15 µM P; HP, 500 µM P. Except for control, all the roots were inoculated with C. parasiticum (see Materials and Methods for details). The solid barrier eliminated root contact and exudates movement, the nylon mesh (30 µm) barrier prevented root intermingling for the two species while permitting root exudates exchange, and no root barrier permitted roots and exudates to completely interact. Each bar represents the mean of three replicates ± SE. Figure E. HPLC scan of soybean and/or maize root exudates. (a and b) HPLC scan of soybean root exudates, (c and d) HPLC scan of maize root exudates, (e and f) HPLC scan of the root exudates from maize/soybean intercropping, (g) HPLC analysis of [file pone.0095031.s001.pdf]
