# Supplementary material for: Root Interactions in a Maize/Soybean Intercropping System Control Soybean Soil-Borne Disease, Red Crown Rot
Source: PLoS One. 2014 May 8;9(5):e95031. doi: 10.1371/journal.pone.0095031 (PMC4014482; doi:10.1371/journal.pone.0095031)
Supplement: File S2 — Supporting tables. Table S1. Real time PCR primers designed for this study. Table S2. AMF colonization rate and nodule number of soybean in the field. (DOC) [file pone.0095031.s002.doc]

**Table S1. Real time PCR primers designed for this study.**

| Gene | Forward/reverse primers | Target sequence^a^ | Ta (℃)^b^ | Amplicon (bp) | Description |
| --- | --- | --- | --- | --- | --- |
| *PR1* | TGATGTTGCCTACGCTCAAG | AF136636 | 61 | 137 | PR1a precursor |
|  | AAGCAGCAACCGTATCATCC |  |  |  |  |
| *PR2* | GTCTCCTTCGGTGGTAGTG | M37753 | 57 | 104 | Beta 1-3 Endoglucanase |
|  | ACCCTCCTCCTGCTTTCTC |  |  |  |  |
| *PR3* | GCACTTGGTCTGGATTTG | AF202731 | 53 | 115 | Chitinase class I |
|  | GGCTTGATGGCTTGTTTC |  |  |  |  |
| *PR4* | GCTTGCGGGTGACAAATAC | Z11977 | 57 | 96 | Wound-induced protein |
|  | ACACTCCCACGTCCAAATC |  |  |  |  |
| *PR10* | GCCCAGGAACCATCAAGAAG  CGCTGTAGCTGTATCCCAAG | AJ289155 | 58 | 108 | Stress-induced  ribonuclease-like protein |
| *PR12* | CATGGACAAGGCACGATTTGG | BU964598 | 62 | 108 | Defensin precursor |
|  | AACCGATGGCTCTTTGACTCAC |  |  |  |  |
| *PAL* | GTGCAAGGGCTGCTTATG  CCCAGTCCCTAATTCCTCTC | X52953 | 57 | 107 | Phenylalanine ammonia-lyase |
| *PPO* | GGGTTGGTGCTGCTGATAAG | EF158428 | 62 | 100 | Polyphenol oxidase |
|  | CGATCCGAGTTCGTGTGATG |  |  |  |  |

^a^ NCBI accession number of *Glycine max* gene. ^b^ Primer annealing temperature.

**Table S2. AMF colonization rate and nodule number of soybean in field experiments.**

|  | P level | Healthy plant | | |  | Infected plant | | |
| --- | --- | --- | --- | --- | --- | --- | --- | --- |
|  |  | MS | ISC1 | ISC2 |  | MS | ISC1 | ISC2 |
| Nodule | LP | 49.50±3.07Aa | 37.75±5.44Aa | 41.75±6.91Aa |  | 29.75±4.33Aa | 22.50±2.40Aa | 25.50±2.40Aa |
| (#/plant) | HP | 61.75±8.21Aa | 42.00±5.87Ab | 32.25±5.54Ab |  | 24.50±1.55Aa | 23.50±3.30Aa | 17.50±2.40Aa |
|  |  |  |  |  |  |  |  |  |
| AMF (%) | LP | 48.89±1.92Aa | 51.68±5.36Aa | 54.28±2.55Aa |  | 47.40±3.30Aa | 50.42±3.92Aa | 53.26±2.71Aa |
|  | HP | 44.63±1.64Aa | 45.99±3.15Aa | 48.83±2.39Aa |  | 43.68±3.62Aa | 42.56±2.13Aa | 44.83±1.95Ba |

Note: Healthy plants were not infected by *C. parasiticum*; infected plants were infected by *C. parasiticum* with severe necrosis on the subterranean stem and roots, and leaf chlorosis. HP: 80 kg P_2_O_5_ ha^-1^ added as calcium superphosphate; LP: no P fertilizer added. MS: soybean monoculture, ISC1: soybean/maize intercropping with 20 cm spacing; ISC2: soybean/maize intercropping with 5 cm spacing. All the data represent the mean of four replicates ± SE. The same upper or lower case letter after numbers in the same column or row for the same trait indicates no significant difference among cultivation modes or two P levels at the critical value of *P*=0.05.
